# Tissue Plasminogen Activator Use in Cardiac Arrest Secondary to Fulminant Pulmonary Embolism

**DOI:** 10.14740/jocmr2452w

**Published:** 2016-01-26

**Authors:** Tariq Yousuf, Taylor Brinton, Khansa Ahmed, Joy Iskander, Daniel Woznicka, Jason Kramer, Adam Kopiec, Amar R. Chadaga, Kathia Ortiz

**Affiliations:** a Department of Internal Medicine, Advocate Christ Medical Center, Oak Lawn, IL, USA; b Rosalind Franklin University of Medicine and Science, Chicago, IL, USA

**Keywords:** Cardiopulmonary resuscitation, Heart arrest, Pulmonary embolism, Thrombolytic therapy, Tissue plasminogen activator

## Abstract

**Background:**

Tissue plasminogen activator (tPA) is used emergently to dissolve thrombi in the treatment of fulminant pulmonary embolism. Currently, there is a relative contraindication to tPA in the setting of traumatic or prolonged cardiopulmonary resuscitation > 10 minutes because of the risk of massive hemorrhage.

**Methods:**

Our single-center, retrospective study investigated patients experiencing cardiac arrest (CA) secondary to pulmonary embolus. We compared the effectiveness of advanced cardiac life support with the administration of tPA vs. the standard of care consisting of advanced cardiac life support without thrombolysis. The primary endpoint was survival to discharge. Secondary endpoints were return of spontaneous circulation (ROSC), major bleeding, and minor bleeding.

**Results:**

We analyzed 42 patients, of whom 19 received tPA during CA. Patients who received tPA were not associated with a statistically significant increase in survival to discharge (10.5% vs. 8.7%, P = 1.00) or ROSC (47.4% vs. 47.8%, P = 0.98) compared to the control group. We observed no statistically significant difference between the groups in major bleeding events (5.3% in the tPA group vs. 4.3% in the control group, P = 1.00) and minor bleeding events (10.5% in the tPA group vs. 0.0% in the control group, P = 0.11).

**Conclusion:**

This study did not find a statistically significant difference in survival to discharge or in ROSC in patients treated with tPA during CA compared to patients treated with standard therapy. However, because no significant difference was found in major or minor bleeding, we suggest that the potential therapeutic benefits of this medication should not be limited by the potential for massive hemorrhage. Larger prospective studies are warranted to define the efficacy and safety profile of thrombolytic use in this population.

## Introduction

Venous thromboembolism, a condition that includes both deep vein thrombosis and pulmonary embolus, is estimated to affect up to 900,000 patients per year in the United States. Within this group, approximately 60,000 - 100,000 patients die from complications [1]. Fulminant pulmonary embolism can lead to cardiac arrest (CA) in 41% of patients [2]. CA secondary to pulmonary embolus often leads to sudden decompensation and is a major predictor of mortality. The most common presentations of massive pulmonary embolus as a cause of CA are asystole and pulseless electrical activity [3-5]. Once CA has occurred, cardiopulmonary resuscitation (CPR) should be immediately initiated. Among the viable treatment options for fulminant pulmonary embolism, thrombolytic agents have received the most attention because of their availability, rapid administration and action, and theoretical pathophysiologic benefits. Current guidelines for the treatment of non-massive pulmonary embolus involve the use of anticoagulants such as heparin. Tissue plasminogen activator (tPA) is indicated to treat massive pulmonary embolus, a pulmonary embolus causing sustained hypotension, pulselessness, or bradycardia. However, a relative contraindication exists for the use of tPA during traumatic or prolonged (> 10 min) resuscitation efforts.

Our study was designed to examine the efficacy and safety of thrombolytic use in patients experiencing CA as a result of pulmonary embolism.

## Methods

For this single-center, retrospective study, we analyzed the cases of patients diagnosed with a pulmonary embolus from 2010 to 2014 who subsequently experienced CA. We investigated the use of tPA to treat patients having CA because of pulmonary embolus and compared this intervention to the standard of care consisting of advanced cardiac life support (ACLS) without thrombolysis. Our primary endpoint was survival to discharge. Additional endpoints included return of spontaneous circulation (ROSC), major bleeding (defined as life-threatening), and minor bleeding.

Inclusion criteria consisted of the diagnosis of a pulmonary embolus by contrast-enhanced computed tomography, ventilation/perfusion scan, or right ventricular strain demonstrated on echocardiogram and subsequent CA during the same hospital admission. Patients were excluded if they had a diagnosis more likely than pulmonary embolus to explain their CA such as tamponade, pneumothorax, myocardial infarction, or septic shock. The thrombolytic of choice at our institution is 100 mg of intravenous alteplase.

Categorical variables are reported as frequencies and percentages, and the continuous variable is reported as mean and standard deviation. Independent sample *t* tests were used to analyze age. Differences for all other variables were calculated with Chi-square analysis or Fisher exact test if any n was < 5. Analysis was performed using SPSS v.22 (IBM), and statistical significance was determined at P < 0.05.

## Results

Demographic and clinical characteristics of the sample are displayed in Table 1. The mean age for the total sample (n = 42) was 66.5 ± 14.1, and the majority of patients were male (n = 24, 57.1%). Fifteen patients (35.7%) had prior use of anticoagulants, and 21.4% had a current diagnosis of cancer (n = 9). Echocardiogram was the most frequently performed diagnostic procedure (n = 24, 57.1%). No statistically significant differences were observed between the groups for age, sex, prior use of anticoagulants, or current diagnosis of cancer (P values ranged from 0.25 to 0.48). More patients in the tPA group (n = 13, 68.4%) had an echocardiogram than in the control group (n = 11, 47.8%).

**Table 1:** Demographic and Clinical Characteristics of the Total Sample and by tPA Versus Control Group

Variable	Total sample (n = 42)	tPA group (n = 19)	Control group (n = 23)	P value
Age, years (mean ± SD)	66.5 ± 14.1	64.6 ± 14.8	68.0 ± 13.6	0.45
Male sex, n (%)	24 (57.1)	9 (47.4)	15 (65.2)	0.25
Prior use of anticoagulant, n (%)	15 (35.7)	9 (47.4)	6 (26.1)	0.42
Current cancer diagnosis, n (%)	9 (21.4)	5 (26.3)	4 (17.4)	0.48
Diagnostic procedure				
Echocardiogram, n (%)	24 (57.1)	13 (68.4)	11 (47.8)	0.04
Other procedure, n (%)	18 (42.9)	6 (31.6)	12 (52.2)	0.04

tPA: tissue plasminogen activator.

**Figure 1:**
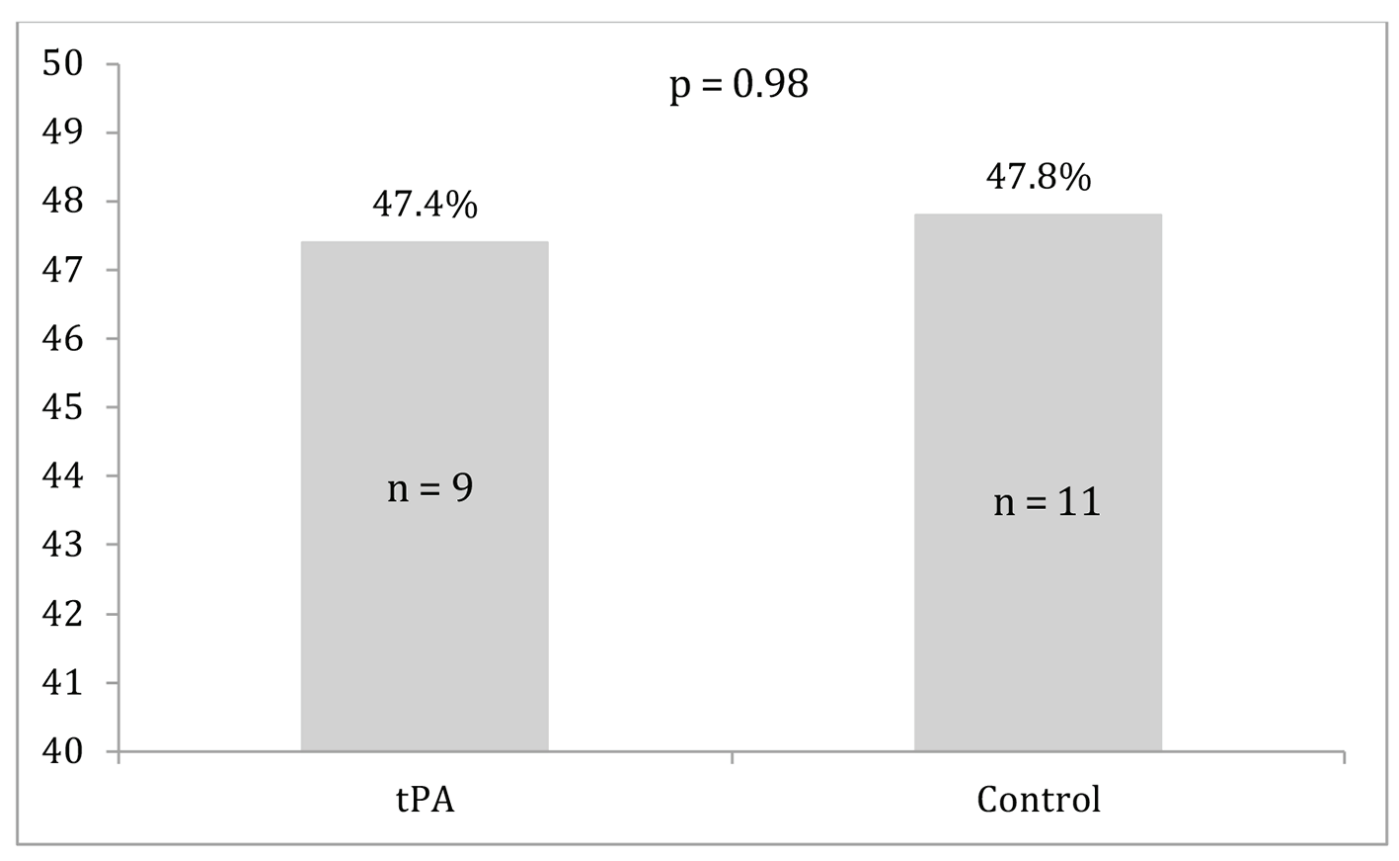
Survival to discharge for the tissue plasminogen activator (tPA) and control groups.

**Figure 2:**
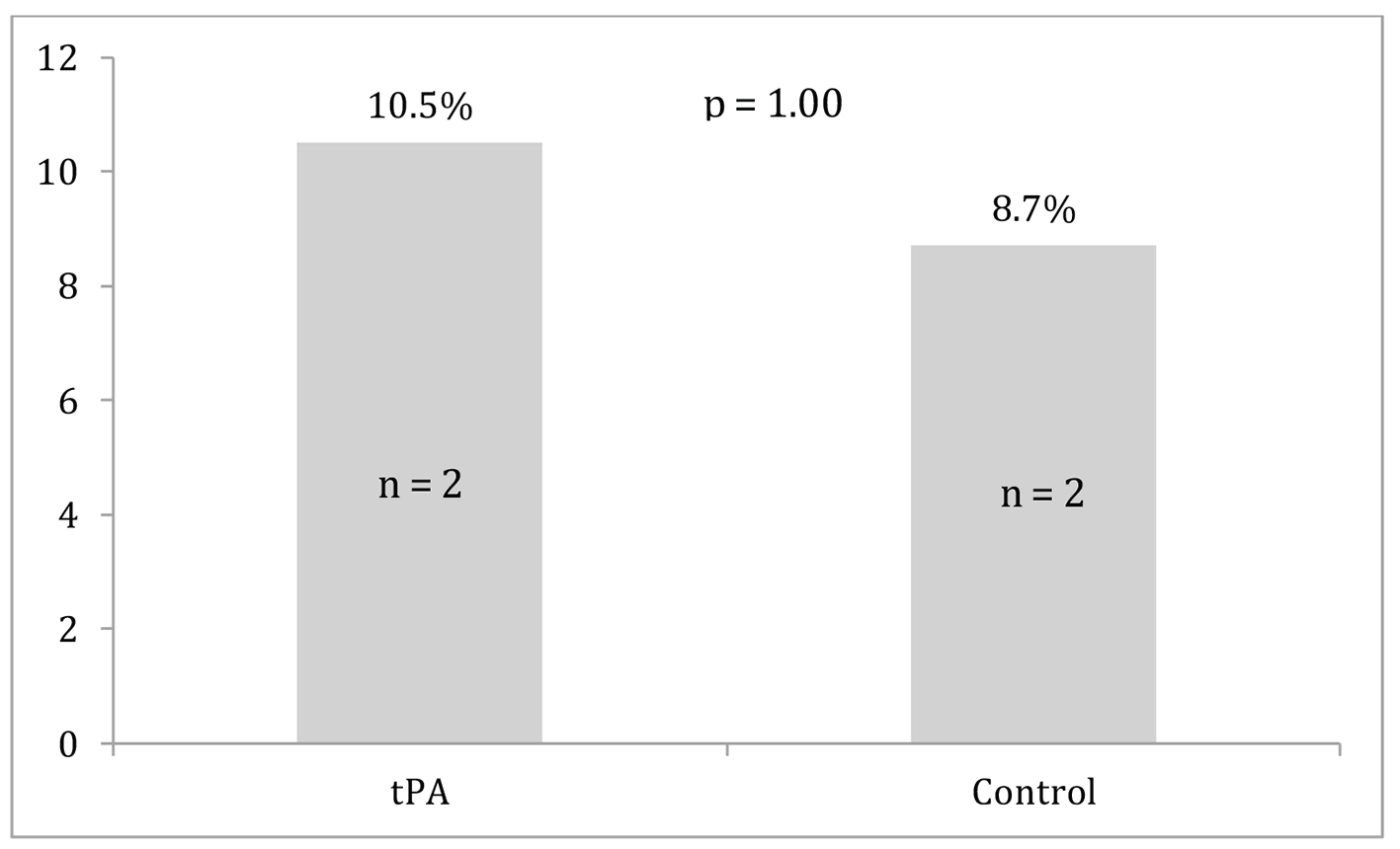
Return of spontaneous circulation for the tissue plasminogen activator (tPA) and control groups.

**Figure 3:**
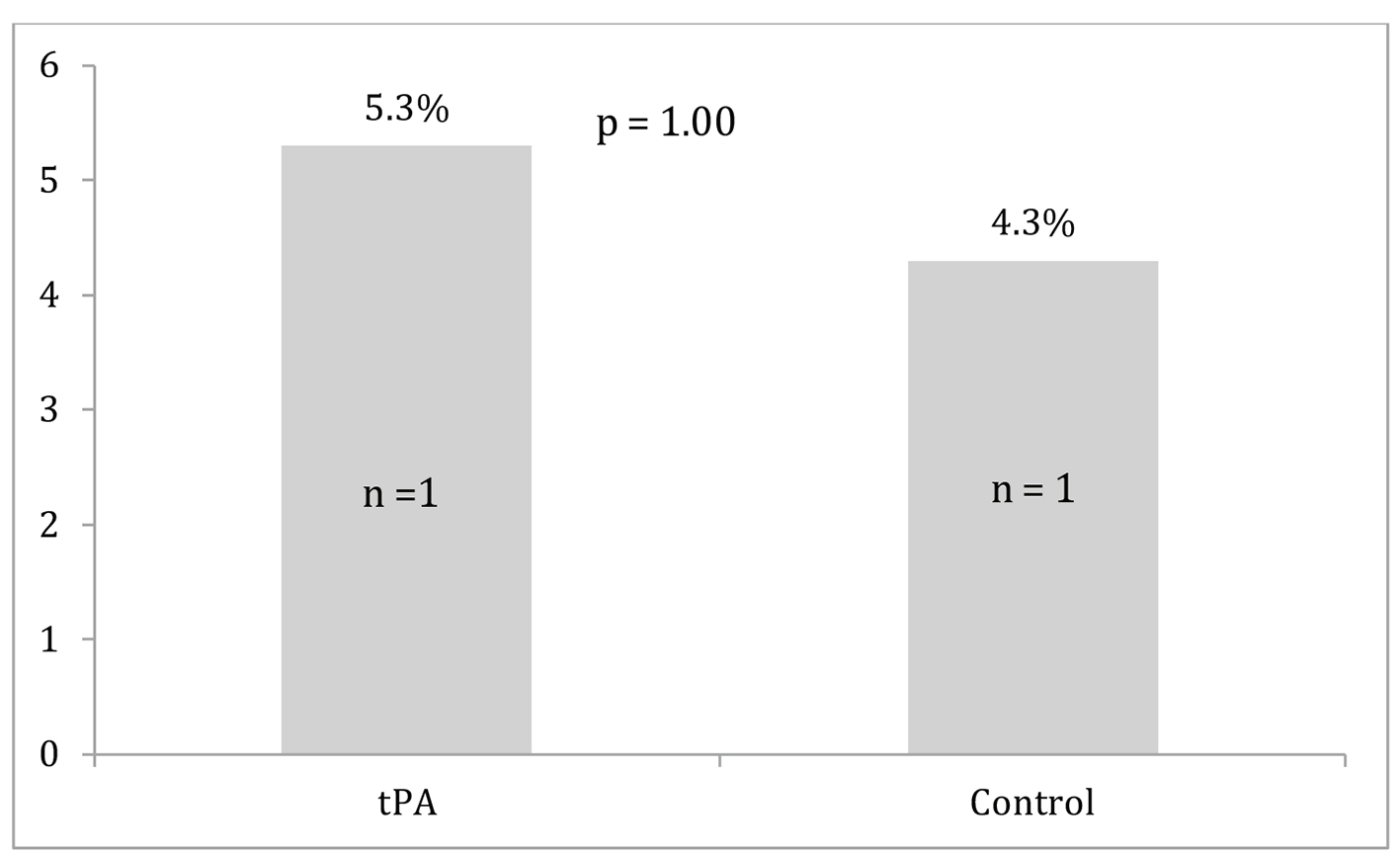
Major bleeding events for the tissue plasminogen activator (tPA) and control groups.

Differences between the two groups in survival to discharge, ROSC, and major bleeding events are displayed in Figures 1-3. Survival to discharge did not differ significantly between groups (10.5% in the tPA group vs. 8.7% in the control group, P = 1.00). No significant difference was noted in ROSC between the two groups (47.4% in the tPA group vs. 47.8% in the control group, P = 0.98). Similarly, major bleeding events did not significantly differ between the groups (5.3% in the tPA group vs. 4.3% in the control group, P = 1.00), and no significant difference was noted in minor bleeding events (10.5% in the tPA group vs. 0.0% in the control group, P = 0.11).

## Discussion

Contraindications for using tPA in patients experiencing CA caused by pulmonary embolus are based on concerns for major bleeding, defined as life-threatening or severe hemorrhage. However, our data showed that intervention with tPA did not significantly increase the rate of major or minor bleeding. Our data also did not show a statistically significant difference between groups in survival or ROSC. These findings are consistent with a review of cases that examined thrombolytic therapy use in the arrest or periarrest setting [2].

We performed a review of the literature on the administration of thrombolytics for patients suspected of having a fulminant pulmonary embolism who received CPR during CA. We searched PubMed and Medline for reports in English from January 2001 to April 2015, using the following keywords: pulmonary embolism, fulminant, CA, CPR, thrombolysis, therapy, prognosis, and complications. We reviewed all works on the subject, including experimental and clinical studies. Table 2 presents our summaries of the case reports we identified on this literature search on this topic [6-9].

**Table 2:** Summary of Case Reports on the Use of Thrombolytics During Cardiopulmonary Resuscitation

Authors	Year	Patient age and sex	History	Thrombolysis regimen	Complications
Zhu et al [6]	2015	33-year-old female	Thrombotic thrombocytopenic purpura	5 mg of rtPA by deep vein catheter and another 45 mg by constant pump infusion	No complications
Toprak et al [7]	2014	55-year-old male	Syncopal episodes and angina on exertion	2 doses of 25 mg rtPA at 5-min intervals directly into the pulmonary artery via a pigtail catheter	No complications
Hsin et al [8]	2014	52-year-old female	Paroxysmal dyspnea	Urokinase 10^6^ U in 100 mL of normal saline infused during a 30-min interval	Non-coagulated blood causing ascites, resolved
Gupta et al [9]	2014	52-year-old male	Unconscious at home	Alteplase at a dose of 0.6 mg/kg during a 2-min interval and a heparin drip	No complications

rtPA: recombinant tissue plasminogen activator.

### Fatal hemorrhage

Bailen et al compiled a comprehensive review of case reports and case series from 1974 to 2000 in 2001 [2]. They found no increase in fatal hemorrhages in patients who received thrombolytics during CA secondary to fulminant pulmonary embolism. Dirican et al reviewed the cases of 22 patients who experienced CA secondary to acute and massive pulmonary embolus between January 2010 and December 2013 at a single center in Turkey [10]. Massive pulmonary embolus was defined as a pulmonary embolus causing hemodynamic instability. Of the 22 patients, 11 received recombinant tPA, and although only four patients survived without sequelae, none of the patients who received tPA had a fatal hemorrhage.

One of the four case reports summarized in Table 2 describes complications from thrombolytics. Hsin et al [8] report ascites caused by non-coagulated blood. The other three cases offer additional evidence that hemorrhage secondary to thrombolytic therapy will likely not be the cause of death of a patient undergoing CA secondary to fulminant pulmonary embolus. Further, given the high mortality rate associated with CA secondary to fulminant pulmonary embolism, it is reasonable to consider thrombolytic therapy an aggressive but ethically justifiable measure in this patient population.

### Survival to discharge and ROSC

Yin et al published a retrospective review of seven patients at the Beijing Chaoyang Hospital who underwent CA with a high clinical suspicion of pulmonary embolus and were administered 50 mg of tPA in a 15-min period after initial CPR was unsuccessful. Five of the seven patients achieved ROSC after thrombolytic therapy, and three of the seven patients were discharged alive. From these cases, Yin et al inferred that thrombolysis may prove to be a successful therapy in patients with presumed pulmonary embolus after initial CPR has been unsuccessful [11].

In 2014, Logan et al published a literature review of retrospective and prospective studies from 1995 to 2012 examining the use of thrombolytic therapy in patients who experienced arrest or periarrest [12]. The retrospective studies in the review generally showed improved outcomes with administration of tPA by demonstrating that the use of thrombolytics improved or had no effect on the ability to achieve ROSC and survival to discharge [12]. The findings of improved ROSC in the tPA groups in some studies were statistically significant, but both studies by Bottinger et al did not show a statistically significant improvement in ROSC. Improved survival to discharge was only statistically significant in three of the five studies that analyzed this endpoint [12]. ROSC can be an indirect measure of success, as earlier achievement can lead to better outcomes. The literature review yielded some extraordinary examples of situations in which thrombolytics provided a mortality benefit [6-9]. However, our review showed that the case reports in Table 2 may be exceptions to the rule, and thrombolytic therapy may need to be used on a case-by-case basis. The success of thrombolytic therapy cannot be measured by mortality benefit alone.

Our review also focused on patients achieving ROSC with tPA administration. Quicker achievement of ROSC may indirectly decrease mortality and morbidity. The difference in ROSC between our two groups was not statistically significant, but Kurkciyan et al [13] showed intriguing evidence that thrombolytic therapy can increase the likelihood of achieving ROSC in patients undergoing CA. Forty-two patients who underwent CA secondary to pulmonary embolus were analyzed, and 21 had received thrombolytic therapy and CPR. While only two patients from the thrombolytic treatment group survived to be discharged compared to one patient in the non-thrombolytic treatment group, 81% of patients receiving thrombolysis achieved ROSC. In contrast, only 33% of patients in the non-thrombolytic treatment group achieved ROSC. Although mortality did not improve, patients receiving thrombolysis showed an increase in spontaneous circulation, indicating that thrombolytic therapy could provide time for further resuscitation efforts during CA.

### Diagnosis of pulmonary embolism

Transesophageal echocardiogram (TEE) is an excellent diagnostic modality because it provides clear images that can help identify pulmonary embolus. TEE also allows clinicians to rule out the possibility of pulmonary embolus while allowing CPR to continue [14]. Commonly, the TEE findings associated with pulmonary embolus are dilation of the right ventricle, right ventricular dysfunction, dilation of the inferior vena cava without collapse during inspiration, and pulmonary hypertension. If these findings are present, the use of thrombolytic therapy could be justified, as thrombolysis can hasten the resolution of pulmonary occlusion and improve right ventricular function [15]. If TEE were to become a standard imaging modality for patients experiencing CA of unknown origin, accurate diagnostic data for a pulmonary embolus could potentially be collected, and clinicians could feel comfortable administering thrombolytics to patients with CA secondary to fulminant pulmonary embolism. The difficulties in obtaining a TEE during CA are availability, operator experience, and the necessity to obtain an airway.

### Study limitations

Our study has several limitations. First, we only analyzed patients from one tertiary academic center, so our ability to generalize is limited. Our results must be interpreted within the context of the management practices followed at our institution. Future studies should be conducted at multiple hospitals to compare institutional differences. Second, we performed our study as a retrospective analysis of data spanning 5 years and obtained a relatively small sample size. Our study was not blinded, so confounding biases cannot be ruled out. A multicenter study may help with variance in operator dependence regarding diagnosis and familiarity with thrombolytic use. Third, we did not follow the survivors 30 days post-discharge to examine long-term outcomes. Last, we did not obtain data on patient comorbidities. Patients with multiple comorbidities may be less likely to survive, to achieve ROSC, and to be free from complications. In addition, the cause of CA can be unclear in patients with multiple comorbidities. To address this ambiguity, our inclusion criteria specified that patients had to be diagnosed with a pulmonary embolus and experience CA during the same hospital admission. This temporal association attempted to directly correlate the CA to the pulmonary embolus. We also excluded any patients who had another more probable diagnosis at the time of CA.

### Conclusion

Our study did not demonstrate a statistically significant difference in our primary endpoint of survival to discharge for patients treated with tPA during CA secondary to pulmonary embolus compared to the standard of care (ACLS without thrombolysis). Similarly, ROSC did not differ significantly between the two groups. However, because our study showed no significant increase in the secondary endpoints of major or minor hemorrhage associated with thrombolytic administration, we suggest that the therapeutic benefits of this medication should not be limited by the potential complication of massive hemorrhage. Some similar studies and case reports have shown thrombolytics to have beneficial effects in the arrest setting. Further investigation is required to fully understand the benefits and limitations of thrombolytic therapy during CA.
